# Dual Quaternions as Constraints in 4D-DPM Models for Pose Estimation

**DOI:** 10.3390/s17081913

**Published:** 2017-08-19

**Authors:** Enrique Martinez-Berti, Antonio-José Sánchez-Salmerón, Carlos Ricolfe-Viala

**Affiliations:** Departamento de Ingeniería de Sistemas y Automática, Instituto de Automática e informática Industrial, Universitat Politècnica de València, València 46022, Spain ; asanchez@isa.upv.es (A.-J.S.-S.); cricolfe@isa.upv.es (C.R.-V.)

**Keywords:** DPM, 4D-DPM, dual quaternions, Kalman filter, polishphere, pose estimation, kinematic constraints

## Abstract

The goal of this research work is to improve the accuracy of human pose estimation using the Deformation Part Model (DPM) without increasing computational complexity. First, the proposed method seeks to improve pose estimation accuracy by adding the depth channel to DPM, which was formerly defined based only on red–green–blue (RGB) channels, in order to obtain a four-dimensional DPM (4D-DPM). In addition, computational complexity can be controlled by reducing the number of joints by taking it into account in a reduced 4D-DPM. Finally, complete solutions are obtained by solving the omitted joints by using inverse kinematics models. In this context, the main goal of this paper is to analyze the effect on pose estimation timing cost when using dual quaternions to solve the inverse kinematics.

## 1. Introduction

Human pose estimation has been extensively studied for many years in computer vision. Many attempts have been made to improve human pose estimation with methods that work mainly with monocular red–green–blue (RGB) images such as [1,2,3,4,5].

With the ubiquity and increased use of depth sensors, methods that use red–green–blue-depth RGB-D imagery are fundamental. One of the methods that used such imagery, and which is currently considered the state of the art for human pose estimation, is Shotton et al. [6], which was commercially developed for the kinect device (Microsoft, Redmond, WA, USA). Shotton’s method allows real-time joint detection for human pose estimation based solely on depth channel. Despite the state-of-the-art performance of [6] and the commercial success of kinect, the many drawbacks of [6] make it difficult to be adopted in any other type of three-dimensional (3D) computer vision system.

Some of the drawbacks of [6] include copyright and licensing issues, which restrict the use and implementation of the algorithm for working on any other devices. Another drawback of the algorithm is the large number of training examples (hundreds of thousands) that are required to train its deep random forest algorithm, and which could make training cumbersome. Another drawback of [6] is that its model is trained only on depth information, and thus discards potentially important information that could be found in the RGB channels and could help approach human poses more accurately. To alleviate these and other drawbacks in [6], we propose a novel approach that takes advantage of both RGB and depth information combined in a multi-channel mixture of parts for pose estimation in single frame images coupled with a skeleton constrained linear quadratic estimator (Kalman filter) that uses the rigid information of a human skeleton to improve joint tracking in consecutive frames. Unlike kinect, our approach makes our model easily trainable even for nonhuman poses. By adding depth information, we increase the time complexity of the proposed method. For this reason, to speed up the proposed method, we reduced the number of points modeled in the proposed method compared with the original deformation part model DPM. Finally, we propose an inverse kinematics method for the inference of the joints not considered initially, which cuts the training time.

The main contribution of our method extends to: (i) a multi-channel mixture of parts model that allows the detection of parts in RGBD images; (ii) a linear quadratic estimator (KF) that employs rigid information and connected joints of human pose; (iii) a model for unsolved joints through inverse kinematics that allows the model to be trained with fewer joints and in less time. In our previous work, [7,8], it is shown that computational cost is too high. This is the reason why in this paper a dual quaternion solution is introduced to improve the computational cost of the previously proposed method. Our results show significant improvements over the state of the art in both the publicly available CAD60 data set and our own data set.

### Related Work

Human pose estimation has been intensely studied for decades in the field of computer vision due to its wide applications. Some of the methods in the literature that attempt to solve this problem date back to the use of pictorial structures (PS) introduced by [9]. More recent methods improve the concept of PS with improved features or inference models, as in [3,10,11,12,13]. Recently, the launch of low-cost RGB-D sensors (e.g., kinect) has further triggered a large amount of research due to their good performance from extra depth information whose intensities depict an inversely proportional relationship between the distance of the objects to the camera. The existing algorithms can be roughly categorized into three groups, i.e., using only RGB sensor, using only Depth sensors, or using both RGB and Depth sensors. Some approaches in the first group are [1,14,15,16,17,18]. Some approaches in the second group are [6,19,20,21,22,23,24,25,26,27,28,29,30,31,32,33,34,35,36,37,38]. Some approaches in the third group are [39,40].

Using RGB sensors, Yang et al. [1] uses a mixtures of parts model based on a robust joint relationships, Sapp et al. [14], in turn, uses a multimodal decomposable model, Bourdev et al. [16] addresses the classic problems of detection, segmentation and pose estimation of people in images with a novel definition of a part, a poselet, and Wang et al. [15] considers part-based models by introducing hierarchical poselets. Ionescu et al. [17] describes automatic 3D human pose reconstruction from monocular images, based on a discriminative formulation with latent segmentation inputs.

Using depth sensor, Shotton et al. [6], which was developed for the kinect algorithm, has become the state of the art for performing human pose estimation that predicts 3D positions of body joints from a single depth image. As mentioned in [26], to capture the human pose efficiently from multi-view video sequences, a sum of Gaussian (SoG) model was developed in [32]. This simple yet effective shape representation provides a differentiable model-to-image similarity function, allowing a fast and accurate full body pose estimation. The SoG model was also used in [33,36,40] for human or hand pose estimation. Extended from SoG, a generalized SoG model (GSoG) was proposed in [34], where it encapsulated fewer anisotropic Gaussians for human shape modeling, and a similarity function between GSoG and SoG was derived in 3D space. Meanwhile, a sum of anisotropic Gaussians (SAG) model [37] shared the similar spirit with GSoG for hand pose estimation, and it provided an overlap measurement between projected SAG and SoG/SAG in 2D image. Although GSoG and SAG based approaches have improved the pose estimation performance with better model adaptability, their similarity functions are specifically designed for different situations/applications. In addition, the clamping function that aims to handle the model intersection problem in previous SoG-based approaches [32,34,36] leads to a discontinuous energy function that could hinder the gradient-based optimization. In [26], inspired by the classical Kernel Correlation-based algorithm [38], generalizes previous SoG-based methods and derives a unified similarity function from the perspective of Gaussian kernel correlation. Ding et al. [26] embeds a kinematical skeleton into the kernel correlation, which enables us to achieve a fast articulated pose estimation.

Using both RGB and depth sensors, object detection has been done using RGB-D with Markov Random Fields (MRFs) and features from both RGB and Depth [39]. Ding et al. [40] defines a method that can capture a broad range of articulated hand motions at interactive rates.

The proposed method uses both RGB and Depth information and a discriminative method using a deformable parts model combined with a generative method using Kalman filter for tracking the human pose.

**Figure 1:**
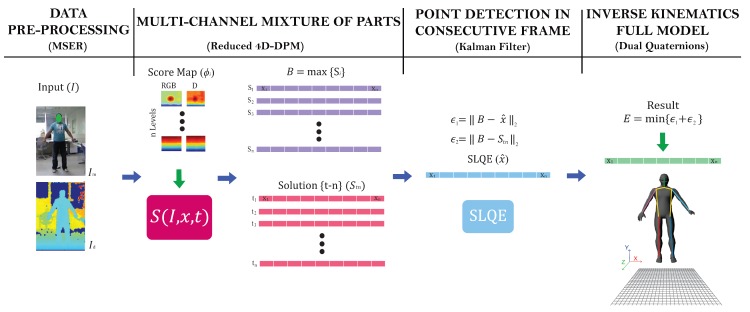
Outline of our method.

We first explain the proposed method, Section 2, using the pre-processing step, Section 2.1, for the depth channels in which the background was removed to improve the accuracy of our algorithm (see Figure 1). Section 2.2 explains the formulation of our four dimensional (4D) mixture of parts model. Section 2.3 explains our structured quadratic linear estimator for correcting joints in consecutive frames. Section 2.4 explains the polisphere model used. Finally, the Section 2.5 describes the strategy to reduce the computational complexity of our proposed method using dual quaternions. Finally, Section 3 shows us the results obtained comparing the proposed method (4D-DPM) with the original method DPM in Section 3.1 and a time complexity analysis in Section 3.2.

## 2. Proposed Method

### 2.1. Data Pre-Processing

As a processing step of RGB channels, we isolate significant foreground areas in these channels from background noise. This is done by removing regions in the depth images that are most unstable to different thresholds that belong to the background. Such a foreground and background template is then transferred to the RGB images to thus remove noise or conflicting object patterns that would confuse foreground and background features in our method, and would hinder detection accuracies.

**Figure 2:**
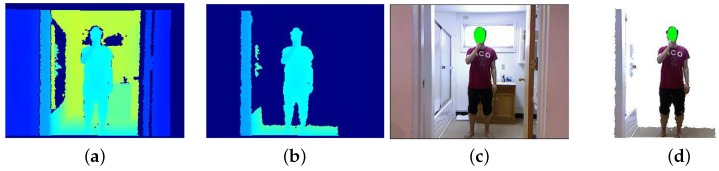
Pre-Processing: (**a**) original depth; (**b**) depth after applying maximally stable extremal regions (MSER); (**c**) original RGB; (**d**) combining image (**c**, **b**).

The intuition behind this approach is that objects or people in the foreground seen through the depth sensor share areas with similar pixel intensities. The reason for this is that the infra red (IR) rays being reflected from the objects in the foreground are reflected more or less at the same time and with the same intensity. Other objects or areas that are much farther away from the IR camera unevenly reflect such rays, and these areas appear noisier and with varying intensities. Figure 2 shows the different intensities reflected from the IR sensor that represents the depth coordinates of the objects.

Due to this property of the pixel intensities in the depth images, our background removal method, which is used for depth and later applied to the RGB images, uses a maximally stable extremal regions (MSER) based approach [41]. These regions are the most stable ones within a range of all possible threshold values being applied to them. A stability score δ of each region in the depth channels is calculated so that $\delta =\frac{\left|\Delta R-R\right|}{\left|R\right|}$, where |R| represents the area of the region in question and Δ represents the intensity variation for the different thresholds. Hence, we remove those MSER regions in which areas are above a *T* threshold. We train the parameters for MSER based on a subset of the training set. We can see in Figure 2 the results from our background subtraction method. Note that most of the noisy pixels in the background have been removed.

### 2.2. Multi-Channel Mixture of Parts

Until recently, Yang and Ramanan’s method [1] has been a state-of-the-art method for pose estimation in monocular images. Yang and Ramanan’s method performs poorly on images that vary from those in its training set, and their method only improves by a small margin even after retraining.

Although there have been other algorithms that have improved Yang and Ramanan’s model, such as [2,3,5], all of these methods, including Yang and Ramanan’s, use a mixture of parts for only the RGB dimension of channels. Conversely, our method uses a multi-channel mixture of parts model that allows us to extend the number of mixtures of parts to the depth dimension of RGBD images.

The depth channel increases time complexity, but this disadvantage has been solved by cutting the number of joints modeled in our 4D-DPM method. On [7,8,42], we can find the main equations changed to introduce the new dimension, depth channel.

### 2.3. Point Detection in Consecutive Frames

To date, we have dealt only with pose estimation for each single frame independently. However, most of the joint movement performed in normal circumstances displays uniform and constant changes of displacement and velocity. Hence, we can use joint velocity and acceleration to predict where joints would most likely be, given their past history. This motion-based prediction could help us validate our frame-based prediction.

One way of predicting joint location based on previous detections is by using a linear quadratic estimator (LQE). Using a simple LQE works well when the joints being tracked are independent of each other and their movement does not correlate. However, in our case, our joints are connected to each other through limbs, which are rigid connections and allow the movement of one joint related to the other one to be connected; e.g., the foot joint movement would be relative to a parent joint like as a knee or hip.

Using the same algorithm as [7,8,42], a Kalman filter is used for tracking the points of interest.

### 2.4. Geometric Model

In the case of improving the results of the Kalman filter, we introduce some restrictions using the geometric model. To do that, we use a polisphere to represent the human body. This representation allows us to detect collisions between the different parts of the body.

**Figure 3:**
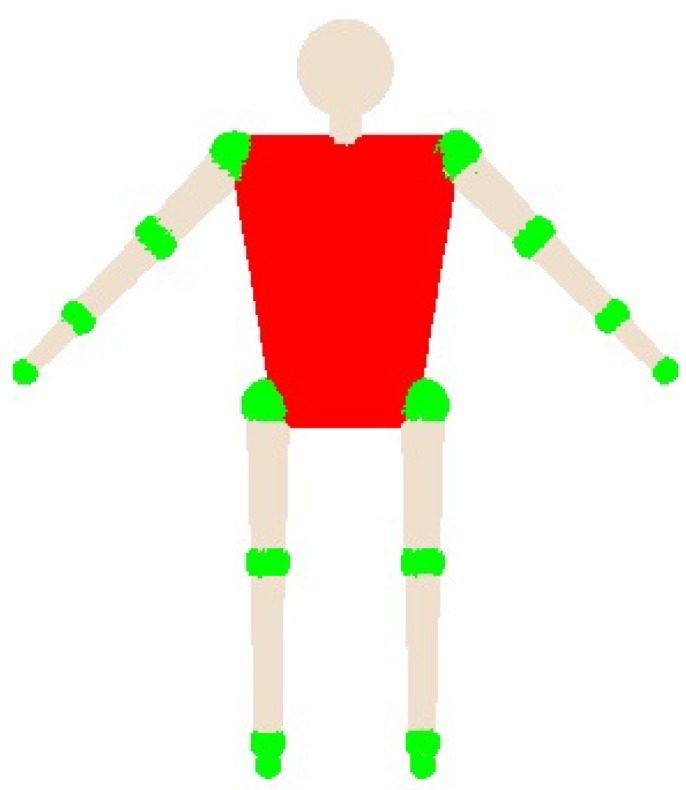
Geometric model using polispheres.

In Figure 3, we can see the geometric model used. Green parts are the principal spheres used and delimit each part of the body.

### 2.5. Model Simplification

The additional depth images included in our formulation add computational cost to our training and testing phases.

In this section, we explain a simplification technique that uses inverse kinematic equations in order to infer shoulder and knee joints. The original DPM model calculates the full body parts with 14 joints. By using inverse kinematics, we can lower that number of points to 10. The joints modeled in our proposed 4D-DPM method were reduced, as were the variables to be predicted with KF.

**Figure 4:**
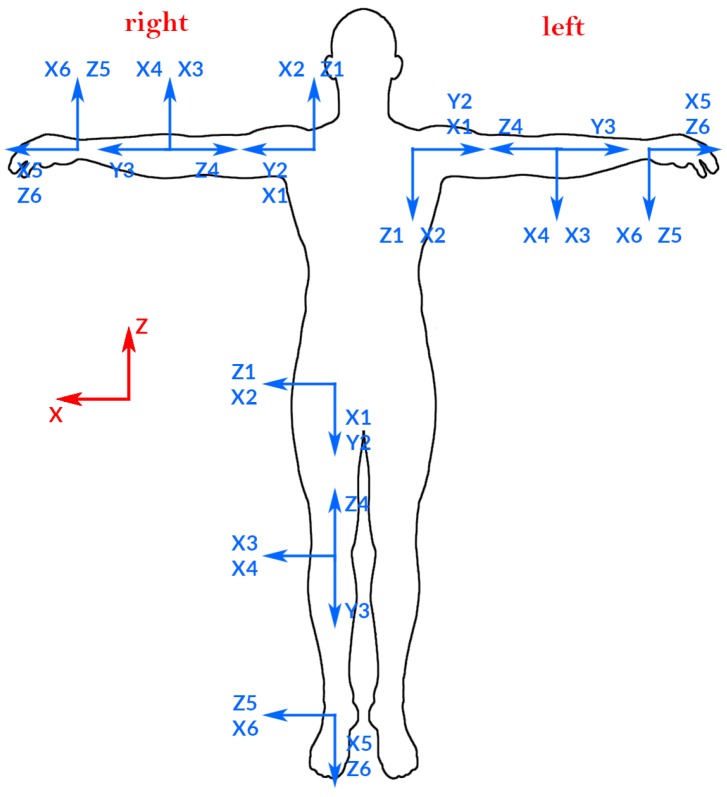
Coordinate systems used.

- **Human body model:** In order to track the human skeleton, we model it as a group of kinematic chains, where each part and joint in the human body corresponds to a link and joint in a kinematic chain. Given the joint positions predicted by the KF, inverse kinematics are used to obtain all of the joints using Dual Quaternions (DQ).
- **State variables:** The human body model is divided into four main kinematic chains (KC) that perform collision detection with their correspondent state variables, in essence: one KC for each arm and one for each leg. Figure 4 shows the state variable for each KC.
- **DQ model:** We use DQ to model each KC. In this sense, we use six joints for each KC for shoulders, hips, hands, and feet (see Figure 4).
- **DH model:** We use the Denavit-Hartenberg (DH) method to obtain the base coordinate system for each joint. After that, we will apply the dual quaternion method. First, we establish the base coordinate system $\left(X_{0},Y_{0},Z_{0}\right)$ at the supporting base with the $Z_{0}$ axis lying along the axis of motion of joint 1. We have four base coordinate systems $\left(X_{0},Y_{0},Z_{0}\right)$, each one located at $\left(X_{1},Y_{1},Z_{1}\right)$ from each KC. Then, we establish a joint axis and align the $Z_{i}$ with the axis of motion of joint i+1.

We also locate the origin of the $i_{th}$ coordinate at the intersection of the $Z_{i}$ and $Z_{i-1}$ or at the intersection of a common normal between the $Z_{i}$ and $Z_{i-1}$. Then, we establish $X_{i}=\pm \left(Z_{i-1}\times Z_{i}\right)/∥Z_{i-1}\times Z_{i}∥$ or along the common normal between the $Z_{i}$ and $Z_{i-1}$ axes when they are parallel. We also assign $Y_{i}$ to complete the right-handed coordinate system. Finally, we find the link and joint parameters: $\theta_{i}$ (angle of the joint with respect to the new axis), $d_{i}$ (offset of joint along the previous axis to the common normal), $a_{i}$ (length of the common normal), and $\alpha_{i}$ (angle of the common normal with respect to the new axis).

For each KC, we have six variable joints $q_{i}$. Each $q_{i}$ is placed on the $z_{i}$ axis in Figure 4. (the left leg in Figure 4 has the same coordinate systems as the right leg.)

Once we have the coordinate systems for each joint, a dual quaternion method is explained. First, we introduce a DQ representation and then we explain the kinematics. A DQ is:

(1) $$\begin{array}{c} \hat{q}=\left({\hat{q}}_{s},{\hat{q}}_{v}\right)\;or\;\hat{q}=q+\varepsilon {\hat{q}}^{0}, \end{array}$$

where ${\hat{q}}_{s}$ is a dual scalar, ${\hat{q}}_{v}$ is a dual vector, *q* and $q^{0}$ are two quaternions and ε is a dual unit. We define the next expressions:

(2) $$\begin{array}{c} \begin{array}{cc} q=(q_{0},q_{1},q_{2},q_{3}) & \hat{q}=\left[\begin{array}{cccc} q_{4} & q_{5} & q_{6} & q_{7}\\ q_{8} & q_{9} & q_{10} & q_{11} \end{array}\right]\\ {\hat{q}}_{s}=q_{s}+\varepsilon {\hat{q}}_{s}^{0} & {\hat{q}}_{v}=q_{v}+\varepsilon {\hat{q}}_{v}^{0}\\ V\left\{q\right\}=q_{v}=\left[q_{1},q_{2},q_{3}\right] & S\left\{q\right\}=q_{s}=q_{0}\\ S\left\{R\left\{\hat{q}\right\}\right\}=q_{s}=q_{4} & S\left\{D\left\{\hat{q}\right\}\right\}=q_{s}^{0}=\left[q_{5},q_{6},q_{7}\right]\\ V\left\{R\left\{\hat{q}\right\}\right\}=q_{v}=q_{8} & V\left\{D\left\{\hat{q}\right\}\right\}=q_{v}^{0}=\left[q_{9},q_{10},q_{11}\right]. \end{array} \end{array}$$

These equations represent different parts of quaternions product: V{q} is a vectorial part, S{q} is a scalar part, $S\{R\left\{\hat{q}\right\}\}$ is a scalar part of real part, $S\{D\left\{\hat{q}\right\}\}$ is a scalar part of dual part, $V\{R\left\{\hat{q}\right\}\}$ is a vectorial part of real part and $V\{D\left\{\hat{q}\right\}\}$ is a vectorial part of dual part.

All the movements of the rigid body in the 3D space, with the exception of pure translation, are equivalent to the screw movements, that is, the rotation on the line together with the translation on the line.

If the line passes over the origin, the movement of screw can be written as:

(3) $$\begin{array}{c} T=\left[\begin{array}{cc} R(\theta ,d) & \frac{\theta }{2\pi }pd\\ 0 & 1 \end{array}\right], \end{array}$$

where R(θ,d) represents the 3×3 rotation matrix on the axis in the direction of the unit vector *d* through an angle θ.

If the axis of the screw movement does not pass over the origin, it can be written as:

(4) $$\begin{array}{c} T=\left[\begin{array}{cc} I_{3x3} & p\\ 0 & 1 \end{array}\right]\left[\begin{array}{cc} R(\theta ,d) & \frac{\theta }{2\pi }pd\\ 0 & 1 \end{array}\right]\left[\begin{array}{cc} I_{3x3} & -p\\ 0 & 1 \end{array}\right]=\left[\begin{array}{cc} R(\theta ,d) & \frac{\theta }{2\pi }pd+\left(I_{3x3}-R\left(\theta ,d\right)\right)p\\ 0 & 1 \end{array}\right]. \end{array}$$

We can represent a screw movement as dual quaternions as follows:

(5) $$\begin{array}{c} \hat{q}=cos\left(\frac{\hat{\theta }}{2}\right)+sin\left(\frac{\hat{\theta }}{2}\right)\hat{d}, \end{array}$$

where $\hat{\theta }=\theta +\epsilon k$ and $\hat{d}=d+\epsilon m$. θ is the rotation of screw angle, and d=[0,d] is the movement of screw axis. The moment of the axis is m=[0,p×d]. The point *p* is in the direction of *d*. And k=d·t.

In a Plücker coordinates, each line can be fully represented by an ordered set of two vectors. The first point is a vector *p* that indicates the position of an arbitrary point on the line, and the second point is the direction vector ***d***, which gives us the direction of the line. The Plücker coordinate can be represented as follows:

(6) $$\begin{array}{c} L_{a}\left(m,d\right), \end{array}$$

where m=p×d is the vector moment of *d* with respect to the reference origin selected.

We can represent one line on Plücker coordiante as ${\hat{l}}_{a}=l_{a}+\epsilon m_{a}$, and we can transform that expression to ${\hat{l}}_{b}^{\prime }=\hat{q}⊙{\hat{l}}_{a}⊙{\hat{q}}^{*}$ using the dual quaternion unit.

The representation of the Plücker coordinates is not minimal since it uses six parameters for the representation of the line. The main advantage of the representation of the Plücker coordinates is that it is homogeneous. $L_{p}\left(m,d\right)$ represents the same line as $L_{p}\left(km,kd\right)$, where k∈ℜ.

**Figure 5:**
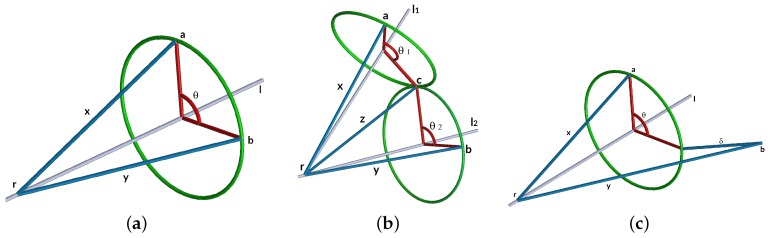
Paden–Kahan sub-problems: (**a**) sub-problem 1; (**b**) sub-problem 2.; (**c**) sub-problem 3.

To solve the forward and inverse kinematics using dual quaternions, we use Paden–Kahan subproblems. We have three sub-problems of Paden–Kahan, and Figure 5 shows graphically the three sub-problems.

To solve the sub-problem 1, we have $y=q⊗x⊗q^{*}$ as the general movement equation, where $\theta =arctan2(S\left\{l⊗x^{{}^{\prime }}⊗y^{{}^{\prime }}\right\},S\left\{x^{{}^{\prime }}⊗y^{{}^{\prime }}\right\})$, $x^{\prime }=x+S\left\{l⊗x\right\}l$, and $y^{\prime }=y+S\left\{l⊗y\right\}l$, l=[0,l] is the director vector of ***l***.

To solve the sub-problem 2, we have $y=q_{1}⊗q_{2}⊗x⊗q_{2}^{*}⊗q_{1}^{*}$ as the general movement equation, z=c−r, z=[0,z]. If $l_{1},l_{2},l_{1}xl_{2}$ are linearly independent, and we have $z=\alpha l_{1}+\beta l_{2}+\gamma \left[0,V\left\{l_{1}⊗l_{2}\right\}\right]$, where $\alpha =\frac{S\left\{l_{1}⊗l_{2}\right\}S\left\{l_{2}⊗x\right\}-S\left\{l_{1}⊗y\right\}}{{\left(S\left\{l_{1}⊗l_{2}\right\}\right)}^{2}-1}$, $\beta =\frac{S\left\{l_{1}⊗l_{2}\right\}S\left\{l_{1}⊗y\right\}-S\left\{l_{2}⊗x\right\}}{{\left(S\left\{l_{1}⊗l_{2}\right\}\right)}^{2}-1}$, and $\gamma =\frac{{\left||x|\right|}^{2}-\alpha^{2}-\beta^{2}-2\alpha \beta S\left\{l_{1}⊗l_{2}\right\}}{||V\left\{l_{1}⊗l_{2}\right\}{\left|\right|}^{2}}$. Then, we can calculate $\theta_{1}$ and $\theta_{2}$ using the sub-problem 1.

To solve the sub-problem 3, we have $||y-q⊗x⊗q^{*}\left|\right|=\left||\gamma |\right|$ as the general movement equation, where $\theta_{0}=arctan2\left(S\left\{l⊗x^{{}^{\prime }}⊗y^{{}^{\prime }}\right\},S\left\{x^{{}^{\prime }}⊗y^{{}^{\prime }}\right\}\right)$; then $\theta =\theta_{0}\pm cos^{-1}\left(\frac{\left|\right|x^{{}^{\prime }}{\left|\right|}^{2}+\left|\right|y^{{}^{\prime }}{\left|\right|}^{2}-\gamma^{{}^{\prime }2}}{2||x^{{}^{\prime }}\left|||\right|y^{{}^{\prime }}\left|\right|}\right)$, where $x^{\prime }=x+S\left\{l⊗x\right\}l$, $y^{\prime }=y+S\left\{l⊗y\right\}l$, $\gamma^{{}^{\prime }2}=\gamma^{2}+\left|S\left\{l⊗\left(a-b\right)\right\}\right|$.

For forward kinematics, given the six variable joints $\left(q_{1},q_{2},q_{3},q_{4},q_{5},q_{6}\right)$, we obtain the coordinates of end effector $\left(x,y,z\right)$ with respect to the base of the KC.

As Equation (1), the transformation operators DQ can be obtained as follows:

(7) $$\begin{array}{c} {\hat{q}}_{i}=\left({\hat{q}}_{S_{i}},{\hat{q}}_{V_{i}}\right)\;or\;{\hat{q}}_{i}=q+\varepsilon {\hat{q}}_{i}^{0}, \end{array}$$

where for prismatic joints $q_{i}=\left[1,0,0,0\right]$ and $q_{i}^{0}=\left[0,q_{1}^{0},q_{2}^{0},q_{3}^{0}\right]$, for revolute joints $q_{i}=\left[\cos\left(\frac{\theta_{i}}{2}\right),\sin\left(\frac{\theta_{i}}{2}\right)d_{i}\right]$ and $q_{i}^{0}=\frac{1}{2}\left(p_{i}-q_{i}⊗p_{i}⊗q_{i}^{*}\right)⊗q_{i}$ or $q_{i}^{0}=\left[0,\sin\left(\frac{\theta_{i}}{2}\right)m_{i}\right]$. In addition, where $d_{i}$ is the rotation axis vector, $m_{i}$ is the vector moment, and $\theta_{i}$ is the angle of rotation and i=1,2,...n.

The general rigid body transformation operation is given by:

(8) $$\begin{array}{c} {\hat{q}}_{1n}={\hat{q}}_{1}⊙{\hat{q}}_{2}⊙{\hat{q}}_{3}⊙\cdots ⊙{\hat{q}}_{n}, \end{array}$$

where ${\hat{q}}_{1n}=q_{1n}+\varepsilon {\hat{q}}_{s}^{0}$. ${\hat{q}}_{1n}$ transform vectors and positions from 1 to *n*.

The orientation and position of the end effector can be found as follows: ${\hat{l}}_{n}=l_{n}+\varepsilon l_{n}^{0}$ and ${\hat{l}}_{n-1}=l_{n}+\varepsilon l_{n-1}^{0}$ are the representations of the Plücker coordinates $n^{th}$ and ${\left(n-1\right)}^{th}$, respectively. We also have ${\hat{l}}_{n}^{{}^{\prime }}=l_{n}^{{}^{\prime }}+\varepsilon l_{n}^{{}^{\prime }0}={\hat{q}}_{1n}⊙{\hat{l}}_{n}⊙{\hat{q}}_{1n}^{*}$, and ${\hat{l}}_{n-1}^{{}^{\prime }}=l_{n-1}^{{}^{\prime }}+\varepsilon l_{n-1}^{{}^{\prime }0}={\hat{q}}_{1n-1}⊙{\hat{l}}_{n-1}⊙{\hat{q}}_{1n-1}^{*}$ are the representations of the Plücker after transformation. The orientation of the end effector is ${\hat{l}}_{6}^{\prime }$. The end effector position can be found as follows:

(9) $$\begin{array}{c} p_{n}=\left(V\left\{R\left\{{\hat{q}}_{1n}⊙{\hat{l}}_{n}⊙{\hat{q}}_{1n}^{*}\right\}\right\}\right)\times \left(V\left\{D\left\{{\hat{q}}_{1n}⊙{\hat{l}}_{n}⊙{\hat{q}}_{1n}^{*}\right\}\right\}\right)\\ +(\left(\left(V\left\{R\left\{{\hat{q}}_{1n-1}⊙{\hat{l}}_{n-1}⊙{\hat{q}}_{1n-1}^{*}\right\}\right\}\right)\times \left(V\left\{D\left\{{\hat{q}}_{1n-1}⊙{\hat{l}}_{n-1}⊙{\hat{q}}_{1n-1}^{*}\right\}\right\}\right)\right)\\ \cdot \left(V\left\{R\left\{{\hat{q}}_{1n}⊙{\hat{l}}_{n}⊙{\hat{q}}_{1n}^{*}\right\}\right\}\right))*\left(V\left\{R\left\{{\hat{q}}_{1n}⊙{\hat{l}}_{n}⊙{\hat{q}}_{1n}^{*}\right\}\right\}\right). \end{array}$$

We can obtain Equation (9) using the intersection of two orthogonal unit line vectors given by:

(10) $$\begin{array}{c} r=d_{b}\times m_{b}+\left(d_{a}\times m_{a}\cdot d_{b}\right)d_{b}, \end{array}$$

(11) $$\begin{array}{c} r=d_{a}\times m_{a}+\left(d_{b}\times m_{b}\cdot d_{a}\right)d_{a}. \end{array}$$

For inverse kinematics, given the coordinates of end effector, $p_{6}$, and the orientation, ${\hat{l}}_{6}^{\prime }$, in Euler parameters, $\left(x,y,z,\phi ,\theta ,\psi \right)$, we can obtain the six variable joints, $\left(q_{1},q_{2},q_{3},q_{4},q_{5},q_{6}\right)$, as we show below.

We have as input parameters:

(12) $$\begin{array}{c} {\hat{q}}_{in}=\left[\begin{array}{c} q_{in}\\ q_{in}^{0} \end{array}\right]=\left[\begin{array}{c} {\hat{l}}_{6}^{{}^{\prime }}\\ p_{6} \end{array}\right], \end{array}$$

where $q_{in}=\left[q_{0},q_{1},q_{2},q_{3}\right]$, end effector orientation, is a real part of dual quaternion ${\hat{q}}_{in}$. In addition, $q_{in}^{0}=\left[q_{0}^{0},q_{1}^{0},q_{2}^{0},q_{3}^{0}\right]$, end effector position, and dual part of dual quaternion ${\hat{q}}_{in}$.

We have then:

(13) $$\begin{array}{c} {\hat{l}}_{6}^{\prime }=R\left\{{\hat{q}}_{16}⊙{\hat{l}}_{6}⊙{\hat{q}}_{16}^{*}\right\}=q_{in},\\ p_{6}=\left(V\left\{R\left\{{\hat{q}}_{16}⊙{\hat{l}}_{6}⊙{\hat{q}}_{16}^{*}\right\}\right\}\right)\times \left(V\left\{D\left\{{\hat{q}}_{16}⊙{\hat{l}}_{6}⊙{\hat{q}}_{16}^{*}\right\}\right\}\right)\\ +(\left(\left(V\left\{R\left\{{\hat{q}}_{15}⊙{\hat{l}}_{5}⊙{\hat{q}}_{15}^{*}\right\}\right\}\right)\times \left(V\left\{D\left\{{\hat{q}}_{15}⊙{\hat{l}}_{5}⊙{\hat{q}}_{15}^{*}\right\}\right\}\right)\right) \end{array}$$

(14) $$\begin{array}{c} \cdot \left(V\left\{R\left\{{\hat{q}}_{16}⊙{\hat{l}}_{6}⊙{\hat{q}}_{16}^{*}\right\}\right\}\right))*\left(V\left\{R\left\{{\hat{q}}_{16}⊙{\hat{l}}_{6}⊙{\hat{q}}_{16}^{*}\right\}\right\}\right)=q_{inV}^{0}. \end{array}$$

An inverse kinematics problem has been solved using the appropriate problems of Paden–Kahan.

Wrist position depends only for the first three joints and wrist orientation depends for the rest of the joints. For this reason, the first joint to calculate is $\theta_{3}$. We define two points, the first point $p_{w}$ allocated on the intersections of axis 5 and 6, and the second point $p_{b}$ on the intersection of axes 1 and 2:

(15) $$\begin{array}{c} \left(V\left\{R\left\{{\hat{q}}_{13}⊙{\hat{l}}_{6}⊙{\hat{q}}_{13}^{*}\right\}\right\}\right)\times \left(V\left\{D\left\{{\hat{q}}_{13}⊙{\hat{l}}_{6}⊙{\hat{q}}_{13}^{*}\right\}\right\}\right)\\ +(\left(\left(V\left\{R\left\{{\hat{q}}_{13}⊙{\hat{l}}_{5}⊙{\hat{q}}_{13}^{*}\right\}\right\}\right)\times \left(V\left\{D\left\{{\hat{q}}_{13}⊙{\hat{l}}_{5}⊙{\hat{q}}_{13}^{*}\right\}\right\}\right)\right)\\ \cdot \left(V\left\{R\left\{{\hat{q}}_{13}⊙{\hat{l}}_{6}⊙{\hat{q}}_{13}^{*}\right\}\right\}\right))*\left(V\left\{R\left\{{\hat{q}}_{13}⊙{\hat{l}}_{6}⊙{\hat{q}}_{13}^{*}\right\}\right\}\right)=q_{inV}^{0}, \end{array}$$

(16) $$\begin{array}{c} \left(V\left\{R\left\{{\hat{q}}_{12}⊙{\hat{l}}_{2}⊙{\hat{q}}_{12}^{*}\right\}\right\}\right)\times \left(V\left\{D\left\{{\hat{q}}_{12}⊙{\hat{l}}_{2}⊙{\hat{q}}_{12}^{*}\right\}\right\}\right)\\ +(\left(\left(V\left\{R\left\{{\hat{q}}_{12}⊙{\hat{l}}_{1}⊙{\hat{q}}_{12}^{*}\right\}\right\}\right)\times \left(V\left\{D\left\{{\hat{q}}_{12}⊙{\hat{l}}_{1}⊙{\hat{q}}_{12}^{*}\right\}\right\}\right)\right)\\ \cdot \left(V\left\{R\left\{{\hat{q}}_{12}⊙{\hat{l}}_{2}⊙{\hat{q}}_{12}^{*}\right\}\right\}\right))*\left(V\left\{R\left\{{\hat{q}}_{12}⊙{\hat{l}}_{2}⊙{\hat{q}}_{12}^{*}\right\}\right\}\right)=q_{b}. \end{array}$$

Doing the subtraction of both points and using the property of the distance between two preservation points by the rigid movements, we obtain sub-problem 3 of Paden–Kahan. The parameters of this sub-problem are:

(17) $$\begin{array}{c} a=\left(V\left\{R\left\{{\hat{l}}_{6}\right\}\right\}\right)\times \left(V\left\{D\left\{{\hat{l}}_{6}\right\}\right\}\right)+\left(\left(\left(V\left\{R\left\{{\hat{l}}_{5}\right\}\right\}\right)\times \left(V\left\{D\left\{{\hat{l}}_{5}\right\}\right\}\right)\right)\cdot \left(V\left\{R\left\{{\hat{l}}_{6}\right\}\right\}\right)\right)*\left(V\left\{R\left\{{\hat{l}}_{6}\right\}\right\}\right)\\ b=\left(V\left\{R\left\{{\hat{l}}_{2}\right\}\right\}\right)\times \left(V\left\{D\left\{{\hat{l}}_{2}\right\}\right\}\right)+\left(\left(\left(V\left\{R\left\{{\hat{l}}_{1}\right\}\right\}\right)\times \left(V\left\{D\left\{{\hat{l}}_{1}\right\}\right\}\right)\right)\cdot \left(V\left\{R\left\{{\hat{l}}_{2}\right\}\right\}\right)\right)*\left(V\left\{R\left\{{\hat{l}}_{2}\right\}\right\}\right), \end{array}$$

and where *l* is the joint 3 and $\delta =q_{in}^{0}-p_{b}$. Using these parameters and using the sub-problem 3, we can find $\theta_{3}$.

If we know $\theta_{3}$ in Equation (15), we can obtain:

(18) $$\begin{array}{c} \left(V\left\{R\left\{{\hat{q}}_{12}⊙{\hat{l}}_{6}⊙{\hat{q}}_{12}^{*}\right\}\right\}\right)\times \left(V\left\{D\left\{{\hat{q}}_{12}⊙{\hat{l}}_{6}⊙{\hat{q}}_{12}^{*}\right\}\right\}\right)\\ +(\left(\left(V\left\{R\left\{{\hat{q}}_{12}⊙{\hat{l}}_{5}⊙{\hat{q}}_{12}^{*}\right\}\right\}\right)\times \left(V\left\{D\left\{{\hat{q}}_{12}⊙{\hat{l}}_{5}⊙{\hat{q}}_{12}^{*}\right\}\right\}\right)\right)\\ \cdot \left(V\left\{R\left\{{\hat{q}}_{12}⊙{\hat{l}}_{6}⊙{\hat{q}}_{12}^{*}\right\}\right\}\right))*\left(V\left\{R\left\{{\hat{q}}_{12}⊙{\hat{l}}_{6}⊙{\hat{q}}_{12}^{*}\right\}\right\}\right)=q_{inV}^{0}, \end{array}$$

where ${\hat{l}}_{6}^{{}^{\prime }}={\hat{q}}_{3}⊙{\hat{l}}_{6}⊙{\hat{q}}_{3}^{*}$ and ${\hat{l}}_{5}^{{}^{\prime }}={\hat{q}}_{3}⊙{\hat{l}}_{5}⊙{\hat{q}}_{3}^{*}$.

With Equation (18), we obtain the sub-problem 2 of Paden–Kahan, where the parameters are:

(19) $$\begin{array}{c} a=\left(V\left\{R\left\{{\hat{l}}_{6}^{{}^{\prime }}\right\}\right\}\right)\times \left(V\left\{D\left\{{\hat{l}}_{6}^{{}^{\prime }}\right\}\right\}\right)+\left(\left(\left(V\left\{R\left\{{\hat{l}}_{5}^{{}^{\prime }}\right\}\right\}\right)\times \left(V\left\{D\left\{{\hat{l}}_{5}^{{}^{\prime }}\right\}\right\}\right)\right)\cdot \left(V\left\{R\left\{{\hat{l}}_{6}^{{}^{\prime }}\right\}\right\}\right)\right)*\left(V\left\{R\left\{{\hat{l}}_{6}^{{}^{\prime }}\right\}\right\}\right), \end{array}$$

and where $l_{1}$ is the joint 1, $d_{1}$; parameter $l_{2}$ is the joint 2, $d_{2}$; value $b=q_{in}^{0}$. With these parameters and the sub-problem 2, we can found $\theta_{1}$ and $\theta_{2}$.

To find the angles of the wrist, we have to consider a new point $p_{i}=p_{6}+\lambda d_{6}$, initial point, allocated over joint axes $d_{6}$. To find the final point $p_{e}$, we need two imaginary axes so that this point is the position of point $p_{i}$ after rotation of angles $\theta_{4}$ and $\theta_{5}$. Point $p_{i}$ is the intersection of imaginary axes. This leads us to:

(20) $$\begin{array}{c} \left(V\left\{R\left\{{\hat{q}}_{45}⊙{\hat{l}}_{8}^{{}^{\prime }}⊙{\hat{q}}_{45}^{*}\right\}\right\}\right)\times \left(V\left\{D\left\{{\hat{q}}_{45}⊙{\hat{l}}_{8}^{{}^{\prime }}⊙{\hat{q}}_{45}^{*}\right\}\right\}\right)\\ +(\left(\left(V\left\{R\left\{{\hat{q}}_{45}⊙{\hat{l}}_{7}^{{}^{\prime }}⊙{\hat{q}}_{45}^{*}\right\}\right\}\right)\times \left(V\left\{D\left\{{\hat{q}}_{45}⊙{\hat{l}}_{7}^{{}^{\prime }}⊙{\hat{q}}_{45}^{*}\right\}\right\}\right)\right)\\ \cdot \left(V\left\{R\left\{{\hat{q}}_{45}⊙{\hat{l}}_{8}^{{}^{\prime }}⊙{\hat{q}}_{45}^{*}\right\}\right\}\right))*\left(V\left\{R\left\{{\hat{q}}_{45}⊙{\hat{l}}_{8}^{{}^{\prime }}⊙{\hat{q}}_{45}^{*}\right\}\right\}\right)=q_{in}^{0}+\lambda d_{6}, \end{array}$$

where ${\hat{l}}_{8}^{{}^{\prime }}={\hat{q}}_{13}⊙{\hat{l}}_{8}⊙{\hat{q}}_{13}^{*}$ and ${\hat{l}}_{7}^{{}^{\prime }}={\hat{q}}_{13}⊙{\hat{l}}_{7}⊙{\hat{q}}_{13}^{*}$. Equation (20) provides the sub-problem 2 of Paden–Kahan. The parameters are:

(21) $$\begin{array}{c} a=\left(V\left\{R\left\{{\hat{l}}_{8}^{{}^{\prime }}\right\}\right\}\right)\times \left(V\left\{D\left\{{\hat{l}}_{8}^{{}^{\prime }}\right\}\right\}\right)+\left(\left(\left(V\left\{R\left\{{\hat{l}}_{7}^{{}^{\prime }}\right\}\right\}\right)\times \left(V\left\{D\left\{{\hat{l}}_{7}^{{}^{\prime }}\right\}\right\}\right)\right)\cdot \left(V\left\{R\left\{{\hat{l}}_{8}^{{}^{\prime }}\right\}\right\}\right)\right)*\left(V\left\{R\left\{{\hat{l}}_{8}^{{}^{\prime }}\right\}\right\}\right), \end{array}$$

and where parameter $l_{1}$ is the imaginary axis 7, $d_{7}$; parameter $l_{2}$ is the imaginary axe 8, $d_{8}$; value $b=q_{in}^{0}+\lambda d_{6}$. With this parameters and the sub-problem 2, we can find $\theta_{4}$ and $\theta_{5}$.

To find the last parameter, $\theta_{6}$, we need a point allowed over the last axis. We define $p_{d}=p_{5}+\lambda d_{5}$. We use two virtual axes to find the point $p_{d}^{\prime }$ that is the position of the point $p_{d}$ after rotation of $\theta_{6}$. Analogously to the above equations and the five angles known, we obtain:

(22) $$\begin{array}{c} \left(V\left\{R\left\{{\hat{q}}_{6}⊙{\hat{l}}_{10}^{{}^{\prime }}⊙{\hat{q}}_{6}^{*}\right\}\right\}\right)\times \left(V\left\{D\left\{{\hat{q}}_{6}⊙{\hat{l}}_{10}^{{}^{\prime }}⊙{\hat{q}}_{6}^{*}\right\}\right\}\right)\\ +(\left(\left(V\left\{R\left\{{\hat{q}}_{6}⊙{\hat{l}}_{9}^{{}^{\prime }}⊙{\hat{q}}_{6}^{*}\right\}\right\}\right)\times \left(V\left\{D\left\{{\hat{q}}_{6}⊙{\hat{l}}_{9}^{{}^{\prime }}⊙{\hat{q}}_{6}^{*}\right\}\right\}\right)\right)\\ \cdot \left(V\left\{R\left\{{\hat{q}}_{6}⊙{\hat{l}}_{10}^{{}^{\prime }}⊙{\hat{q}}_{6}^{*}\right\}\right\}\right))*\left(V\left\{R\left\{{\hat{q}}_{6}⊙{\hat{l}}_{10}^{{}^{\prime }}⊙{\hat{q}}_{6}^{*}\right\}\right\}\right)=q_{in}^{0}+\lambda d_{6}, \end{array}$$

where ${\hat{l}}_{10}^{{}^{\prime }}={\hat{q}}_{15}⊙{\hat{l}}_{10}⊙{\hat{q}}_{15}^{*}$ and ${\hat{l}}_{9}^{{}^{\prime }}={\hat{q}}_{15}⊙{\hat{l}}_{9}⊙{\hat{q}}_{15}^{*}$. Equation (22) allows us to sub-problem 1. The parameters are:

(23) $$\begin{array}{c} a=\left(V\left\{R\left\{{\hat{l}}_{10}^{{}^{\prime }}\right\}\right\}\right)\times \left(V\left\{D\left\{{\hat{l}}_{10}^{{}^{\prime }}\right\}\right\}\right)+\left(\left(\left(V\left\{R\left\{{\hat{l}}_{9}^{{}^{\prime }}\right\}\right\}\right)\times \left(V\left\{D\left\{{\hat{l}}_{9}^{{}^{\prime }}\right\}\right\}\right)\right)\cdot \left(V\left\{R\left\{{\hat{l}}_{10}^{{}^{\prime }}\right\}\right\}\right)\right)*\left(V\left\{R\left\{{\hat{l}}_{10}^{{}^{\prime }}\right\}\right\}\right), \end{array}$$

and where parameter *l* is the imaginary axis 6, $d_{6}$; value $b=q_{in}^{0}+\lambda d_{5}$. With these parameters and the sub-problem 1, we can find $\theta_{6}$.

**Figure 6:**
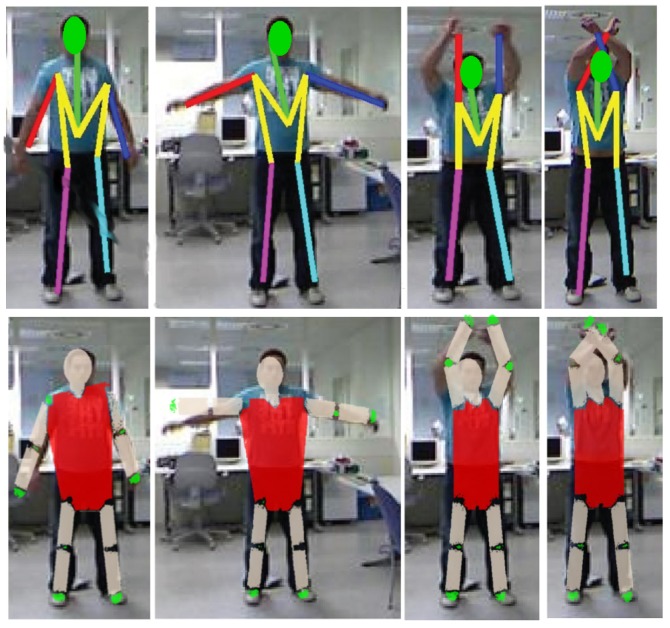
Results of our method after inverse kinematics (IK). The second row shows the model and joints being inferred (elbows and knees).

We use inverse kinematics because we can obtain the base of our KC (shoulders or hips), and where the final effector and the orientation (hands and feet) are; thus, we have these parameters: $\left(x,y,z,\phi ,\theta ,\psi \right)$ and, using inverse kinematics, we obtain the six variable joints,$\left(\theta_{1},\theta_{2},\theta_{3},\theta_{4},\theta_{5},\theta_{6}\right)$, and use them to know where the elbow or knee are located (shown in Figure 6).

## 3. Results

- **3D Camera Calibration:** Our method works with any RGB-D sensor after correct calibration. In our experiments, we use a kinect device and calibrate the intrinsic and extrinsic parameters of the monocular and IR sensors. The calibration system is done similarly to [43] or [44,45].
- **Data sets:** To train and test our method, we use a combination of videos from our own data set and a subset of the publicly available CAD60 data set [46].
- **CAD60 data set:** The original CAD60 data set [46] contains 60 RGB-D videos, four subjects (two male, two female), four different environments (office, bedroom, bathroom and living room) and 12 different activities. This data set was originally created for the activity recognition task [47,48,49]. The size of images is 320 × 240 pixels.
- **Our data set:** It consists of seven videos with only one person on the scene moving his arms and legs. We had almost 1000 frames of people to obtain specific movements, e.g., crossing arms over one’s body, to complement the CAD60 data set. Images were taken indoors in different scenarios. The subject inside the images is a male who wears different clothes. The size of the images is 320 × 240 pixels.

The ground truth of the joints in this data set was obtained by recording predictions from kinect. Thus, in order to make a fair comparison of the predictions from the methods being tested, we provide the videos to our human annotators to manually record the ground truth of the joint positions in the CAD60 data set. Thus, our annotators recorded over 15,000 frames of videos that correspond to 16 videos from the CAD60 data set with different activities and environments. For training and testing purposes, we use two different splits of such annotations. We chose to manually annotate the CAD60 data set because, to our knowledge, there is no RGBD data set with the ground truth of human pose joints. We will also publicly release our annotated videos for the benefit of the research community.

We can find some other data sets using RGB and depth images for pose estimation, but they can not be used in our proposed method due to annotation problems.

**Metrics:** The metrics we use in our different experiments are the probability of a correct kypoint (PCK), the average precision keypoint (APK) and error distance.

**PCK:** The probability of a correct keypoint (PCK) was introduced by Yang and Ramanan [1]. Given the bounding box, a pose estimation algorithm must report back the keypoint locations for body joints. The overlap between the keypoint bounding boxes was measured, which can suffer from quantization artifacts for small bounding boxes. A keypoint is considered correct if it lies within α·max(h,w) of the ground truth bounding box, where *h* corresponds to the height and *w* to the width of the corresponding bounding box. α is a parameter that controls the relative threshold to consider the correctness of the keypoint.

**APK:** In a real system, however, one has no access to annotated bounding boxes at the test time, and one must also address the detection problem. One can cleanly combine the two problems by thinking of body parts (or rather joints) as objects to be detected, and evaluate object detection accuracy with a precision–recall curve. The average precision keypoint is another metric introduced by Yang and Ramanan [1], where, unlike PCK, it penalizes false-positives. Correct keypoints are also determined through the α·max(h,w) relationship.

**Error distance:** This metric calculates the distance between the results and the correct labeled point. To do this, we calculate the distance error between the predicted result and the ground truth location. For each joint, we obtain an error score that is the mean value calculated from all of the frames.

### 3.1. Quantitative Results

Table 1 compares our results with Yang and Ramanan’s [1] original method (Yang*) trained with the same images that we used to train our proposed method (P. Method*). Observing the results obtained in Table 1, and by comparing our proposed method with the original DPM, trained both with the same range of images and tested with the same range of images, but a different one of trained images, we have improved the results with the proposed method by adding depth information, a Kalman filter and using Denavit–Hartenberg (DH), in order to cut the number of points modeled in the DPM. Observing the results in Table 1, and independently of the data set used to test or train parts, our proposed method obtains better solutions. This means that the results can be repeatable with different data sets.

Table 1 shows the results using KF and DH. , and using DQ will obtain the same results in accuracy as using DH. For this reason, a table comparing the original DPM model with our proposed method is not shown. We discuss in the next section the difference between DH and DQ.

### 3.2. Time Complexity Analysis

For our experiments, we use a system based on Windows 7 (Microsoft, Redmond, WA, USA) with 64 bits and 4 GB RAM. The processor used is Inter Core Quad 2.33 GHz (Intel Corporation, Santa Clara, CA, USA). We calculate for each frame the average time taken for the proposed algorithm to process the frame. The images used have 320×240 pixels.

In our previous work, we used (DH) kinematics instead of dual quaternions. Dual quaternions are faster than a transformation matrix used in DH and do not have singularities in their solutions.

**Figure 7:**
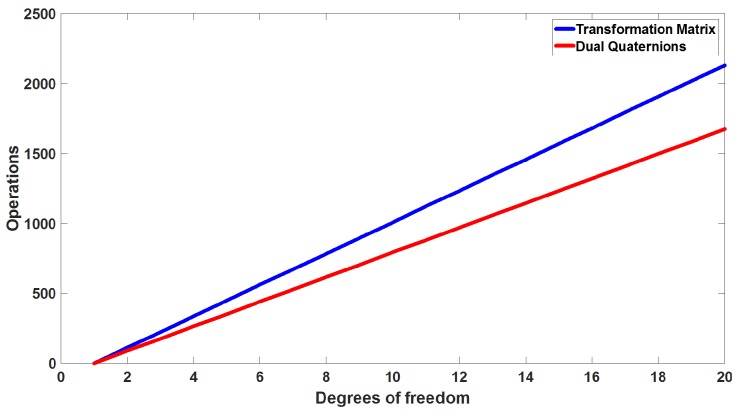
Comparing the number of operations between Denavit–Hartemberg and dual quaternions.

Table 2 shows the number of operations for one degree of freedom, for *n* degrees of freedom, we have on DH 64(n−1) products operations and 48(n−1) between sums and subtraction operations, while, for DQ. we have 48(n−1) products operations and 40(n−1) between sums and subtractions operations. Figure 7 shows how many operations we need for each degree of freedom added.

**Figure 8:**
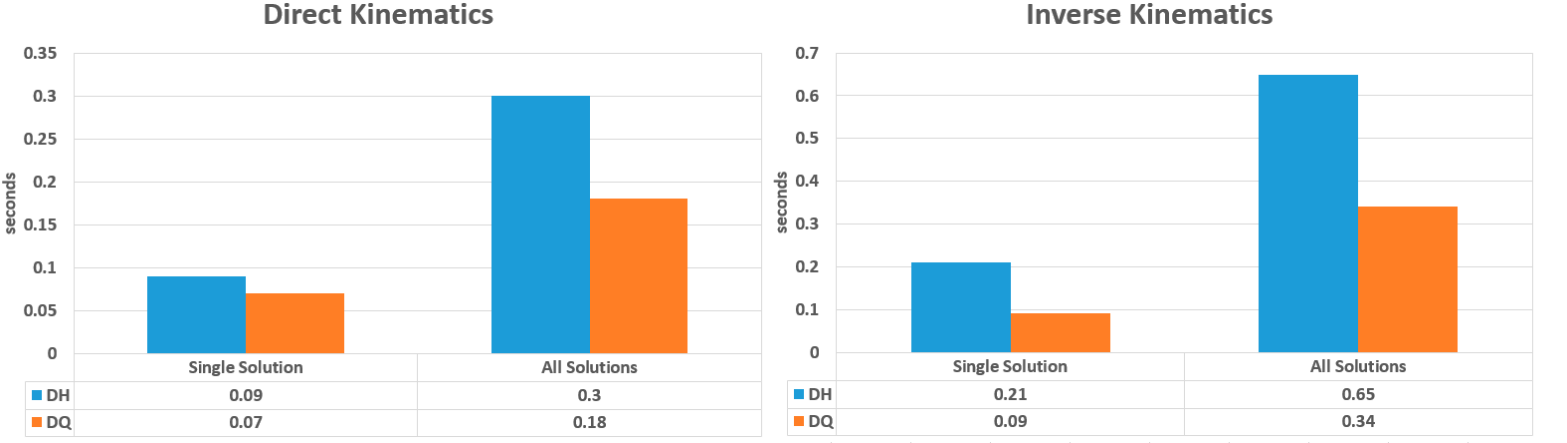
Computational time used.

Figure 8 shows the time needed to make the operations. We can see that DQ is faster than DH; for this reason, we opted to use DQ.

All of these comparisons about computational cost using dual quaternions are for one kinematic chain. In our proposed method, we are using four kinematic chains for which we have to multiply these results by 4.

Finally, the computational cost of the proposed method is 6.85 s and the original DPM method takes 9.21 s.

## 4. Conclusions

In this paper, we have presented a 4D-DPM model using RGB-D information to improve accuracy and timing cost. We use MSER for foreground subtraction. We use dual quaternions to reduce the number of points of interest inside the imagery. We use a polisphere to draw the results and detect collisions between the different parts of the body. All of this allows us to reduce the time complexity during the training part using a smaller fraction of training samples.

## Figures and Tables

**Table 1:** Experimental comparisons with the state-of-the-art methods on our proposed data set. The probability of a correct kypoint (PCK) and the average precision keypoint (APK) metrics are expressed on %. Error is expressed in pixels.

Model	Metric	Head	Shoulders	Wrist	Hip	Ankle	Avg
Yang* [1]	APK	91.20	92.30	82.70	86.60	83.50	87.26
	PCK	91.50	89.00	85.80	89.90	83.80	88.00
	Error	8.17	8.81	10.87	9.37	11.59	9.76
P. Method* with KF with DH	APK	**97.50**	**98.30**	**92.20**	**94.70**	**94.00**	**95.34**
	PCK	**96.40**	**95.20**	**93.70**	**96.50**	**94.20**	**95.20**
	Error	**5.82**	**5.71**	**7.43**	**6.37**	**6.61**	**6.38**

**Table 2:** Number of operations between Denavit–Hartenberg and dual quaternions.

Method	Memory	Products	Sum/Subtract	Total
Homogeneous Matrix	16	64	48	112
Dual Quaternions	8	48	40	88

## Abbreviations

The following abbreviations are used in this manuscript:

PS	Pictorial Structures
MRFs	Markov Random Fields
DPM	Deformable Parts Model
MSER	Maximally Stable Extremal Regions
PCK	Probability of a Correct Kypoint
APK	Average Precision Keypoint
KF	Kalman Filter
DQ	Dual Quaternions
KC	Kinematic Chains

## References

[1] Yang Y., Ramanan D. (2013). Articulated human detection with flexible mixtures of parts. IEEE Trans. Pattern Anal. Mach. Intell..

[2] Wang F., Li Y. Beyond physical connections: Tree models in human pose estimation. Proceedings of the 2013 IEEE Conference on Computer Vision and Pattern Recognition (CVPR).

[3] Pishchulin L., Andriluka M., Gehler P., Schiele B. Poselet conditioned pictorial structures. Proceedings of the 2013 IEEE Conference on Computer Vision and Pattern Recognition (CVPR).

[4] Toshev A., Szegedy C. Deeppose: Human pose estimation via deep neural networks. Proceedings of the 2014 IEEE Conference on Computer Vision and Pattern Recognition (CVPR).

[5] Ramakrishna V., Munoz D., Hebert M., Bagnell J.A., Sheikh Y. (2014). Pose Machines: Articulated Pose Estimation via Inference Machines. Computer Vision–ECCV 2014.

[6] Shotton J., Girshick R., Fitzgibbon A., Sharp T., Cook M., Finocchio M., Moore R., Kohli P., Criminisi A., Kipman A. (2013). Efficient human pose estimation from single depth images. IEEE Trans. Pattern Anal. Mach. Intell..

[7] Martinez E., Nina O., Sanchez A., Ricolfe C. Optimized 4D-DPM for Pose Estimation on RGBD Channels using polisphere models. Proceedings of the 12th International Joint Conference on Computer Vision, Imaging and Computer Graphics Theory and Applications.

[8] Martinez E., Sanchez-Salmeron A.J., Ricolfe-Viala C. (2017). 4D-DPM model for pose estimation using Kalman filter constraints. Int. J. Adv. Robot. Syst..

[9] Fischler M.A., Elschlager R.A. (1973). The representation and matching of pictorial structures. IEEE Trans. Comput..

[10] Eichner M., Ferrari V. Better appearance models for pictorial structures. Proceedings of the British Machine Vision Conference (BMVC).

[11] Andriluka M., Roth S., Schiele B. Pictorial structures revisited: People detection and articulated pose estimation. Proceedings of the 2009 IEEE Conference on Computer Vision and Pattern Recognition (CVPR).

[12] Huang C.M., Chen Y.R., Fu L.C. (2014). Visual tracking of human head and arms using adaptive multiple importance sampling on a single camera in cluttered environments. IEEE Sens. J..

[13] Ning X., Guo G. (2013). Assessing spinal loading using the kinect depth. IEEE Sens. J..

[14] Sapp B., Taskar B. Modec: Multimodal decomposable models for human pose estimation. Proceedings of the 2013 IEEE Conference on Computer Vision and Pattern Recognition (CVPR).

[15] Wang Y., Tran D., Liao Z., Forsyth D. (2012). Discriminative hierarchical part-based models for human parsing and action recognition. J. Mach. Learn. Res..

[16] Bourdev L., Malik J. Poselets: Body part detectors trained using 3d human pose annotations. Proceedings of the 2009 IEEE 12th International Conference on Computer Vision.

[17] Ionescu C., Li F., Sminchisescu C. Latent structured models for human pose estimation. Proceedings of the 2011 IEEE International Conference on Computer Vision (ICCV).

[18] Gkioxari G., Arbeláez P., Bourdev L., Malik J. Articulated pose estimation using discriminative armlet classifiers. Proceedings of the 2013 IEEE Conference on Computer Vision and Pattern Recognition (CVPR).

[19] Grest D., Woetzel J., Koch R. (2005). Nonlinear body pose estimation from depth images. Pattern Recognition.

[20] Plagemann C., Ganapathi V., Koller D., Thrun S. Real-time identification and localization of body parts from depth images. Proceedings of the 2010 IEEE International Conference on Robotics and Automation (ICRA).

[21] Helten T., Baak A., Bharaj G., Muller M., Seidel H.P., Theobalt C. Personalization and Evaluation of a Real-Time Depth-Based Full Body Tracker. Proceedings of the 2013 International Conference on 3D Vision.

[22] Baak A., Müller M., Bharaj G., Seidel H.P., Theobalt C. (2013). A data-driven approach for real-time full body pose reconstruction from a depth camera. Consumer Depth Cameras for Computer Vision.

[23] Spinello L., Arras K.O. People detection in RGB-D data. Proceedings of the 2011 IEEE Intelligent Robots and Systems (IROS).

[24] Ganapathi V., Plagemann C., Koller D., Thrun S. Real time motion capture using a single time-of-flight camera. Proceedings of the 2010 IEEE Conference Computer Vision and Pattern Recognition (CVPR).

[25] Ye M., Yang R. Real-time simultaneous pose and shape estimation for articulated objects using a single depth camera. Proceedings of the IEEE Conference on Computer Vision and Pattern Recognition (CVPR).

[26] Ding M., Fan G. (2016). Articulated and Generalized Gaussian Kernel Correlation for Human Pose Estimation. IEEE Trans. Image Process..

[27] Ganapathi V., Plagemann C., Koller D., Thrun S. Real-time human pose tracking from range data. Proceedings of the 12th European Conference on Computer Vision (ECCV).

[28] Ganapathi V., Plagemann C., Koller D., Thrun S. Real time motion capture using a single time-of-flight camera. Proceedings of the 2010 IEEE Conference on Computer Vision and Pattern Recognition (CVPR).

[29] Baak A., Muller M., Bharaj G., Seidel H., Theobalt C. A datadriven approach for real-time full body pose reconstruction from a depth camera. Proceedings of the 2011 IEEE International Conference on Computer Vision (ICCV).

[30] Ye M., Wang X., Yang R., Ren L., Pollefeys M. Accurate 3D pose estimation from a single depth image. Proceedings of the 2011 IEEE International Conference on Computer Vision (ICCV).

[31] Wei X., Zhang P., Chai J. (2012). Accurate realtime full-body motion capture using a single depth camera. ACM Trans. Graph..

[32] Stoll C., Hasler N., Gall J., Seidel H., Theobalt C. Fast articulated motion tracking using a sums of Gaussians body model. Proceedings of the 2011 IEEE International Conference on Computer Vision (ICCV).

[33] Ding M. (2014). Fast human pose tracking with a single depth sensor using sum of Gaussians models. Adv. Visual Comput..

[34] Ding M., Fan G. Generalized sum of Gaussians for real-time human pose tracking from a single depth sensor. Proceedings of the 2015 IEEE Winter Conference on Applications of Computer Vision (WACV).

[35] Taylor J., Shotton J., Sharp T., Fitzgibbon A. The Vitruvian manifold: Inferring dense correspondences for one-shot human pose estimation. Proceedings of the 2012 IEEE Conference on Computer Vision and Pattern Recognition (CVPR).

[36] Kurmankhojayev D., Hasler N., Theobalt C. (2013). Monocular pose capture with a depth camera using a Sums-of-Gaussians body model. Pattern Recognit..

[37] Sridhar S., Rhodin H., Seidel H., Oulasvirta A., Theobalt C. Real-time hand tracking using a sum of anisotropic Gaussians model. Proceedings of the International Conference on 3D Vision (3DV).

[38] Tsin Y., Kanade T. (2004). A correlation-based approach to robust point set registration. European Conference on Computer Vision.

[39] Lai K., Bo L., Ren X., Fox D. Detection-based object labeling in 3D scenes. Proceedings of the 2012 IEEE International Conference on Robotics and Automation (ICRA).

[40] Sridhar S., Oulasvirta A., Theobalt C. Interactive markerless articulated hand motion tracking using RGB and depth data. Proceedings of the International Conference on Computer Vision (ICCV) 2013.

[41] Matas J., Chum O., Urban M., Pajdla T. (2004). Robust wide-baseline stereo from maximally stable extremal regions. Image Vis. Comput..

[42] Martinez E., Sanchez A., Ricolfe C., Nina O. (2016). Human Pose Estimation for RGBD Imagery with Multi-Channel Mixture of Parts and Kinematic Constraints. WSEAS Trans. Comput..

[43] Berti E.M., Salmerón A.J.S., Benimeli F. (2012). Human-Robot Interaction and Tracking Using low cost 3D Vision Systems. Romanian J. Tech. Sci. Appl. Mech..

[44] Ricolfe C., Sanchez A., Martinez E. (2011). Calibration of a wide angle stereoscopic system. Opt. Lett..

[45] Ricolfe C., Sanchez A., Martinez E. (2012). Accurate calibration with highly distorted images. Appl. Opt..

[46] Sung J., Ponce C., Selman B., Saxena A. (2011). Human activity detection from RGBD images. Plan Act. Intent Recognit..

[47] Wang J., Liu Z., Wu Y. (2014). Learning actionlet ensemble for 3D human action recognition. Human Action Recognition with Depth Cameras.

[48] Shan J., Akella S. 3D Human Action Segmentation and Recognition using Pose Kinetic Energy. Proceedings of the 2014 IEEE Workshop on Advanced Robotics and its Social Impacts (ARSO).

[49] Faria R.D., Premebida C., Nunes U. A Probalistic Approach for Human Everyday Activities Recognition using Body Motion from RGB-D Images. Proceedings of the 2014 RO-MAN: 23rd IEEE International Symposium on Robot and Human Interactive Communication.

